# Zoonotic multidrug-resistant microorganisms among small companion animals in Germany

**DOI:** 10.1371/journal.pone.0208364

**Published:** 2018-12-07

**Authors:** Ursula Kaspar, Alexa von Lützau, Andreas Schlattmann, Uwe Roesler, Robin Köck, Karsten Becker

**Affiliations:** 1 Institute of Medical Microbiology, University Hospital Münster, Münster, Germany; 2 Institute for Animal Hygiene and Environmental Health, FU Berlin, Department of Veterinary Medicine, Berlin, Germany; University Medical Center Groningen, NETHERLANDS

## Abstract

Antimicrobial multidrug-resistant microorganisms (MDRO) can be transmitted between companion animals and their human owners. Aim of this study was to determine the prevalence of extended spectrum beta-lactamase-producing *Enterobacteriaceae* (ESBL-E) and *Staphylococcus aureus* including methicillin-resistant *S*. *aureus* (MRSA) in different companion animal species. Dogs (n = 192), cats (n = 74), and rabbits (n = 17), treated in a veterinary practice and hospital or living in an animal shelter and private households, were sampled. All facilities were located in a region characterized by a high density of pig production. Nasal, buccal and perianal swabs were enriched and cultured on solid chromogenic selective media. A subgroup of 20 animals (13 dogs, 3 cats, 4 rabbits) was analyzed for the presence of staphylococci other than *S*. *aureus*. Amongst all animals (n = 283), twenty dogs (10.4%) and six cats (8.1%) carried *S*. *aureus*. MRSA was found in five dogs (2.6%) and two cats (2.7%). Isolates were of *spa* types t011, t034, t108 (all *mecA*-positive, ST398), and t843 (*mecC*-positive, ST130), typical for livestock-associated (LA)-MRSA. Except for one dog, MRSA-positive animals did not have direct contact to husbandry. ESBL-*Escherichia coli* (*bla*_CTX-M_/*bla*_TEM_/*bla*_SHV_ genes) were present in seven dogs (3.6%), one cat (1.4%) possessed a cefotaxime-resistant *Citrobacter freundii* isolate *(bla*_TEM_/*bla*_CMY-2_ genes). MDRO carriage was associated with animals from veterinary medical settings (p<0.05). One dog and one rabbit carried methicillin-resistant coagulase-negative staphylococci. The exclusive occurrence of MRSA lineages typically described for livestock stresses the impact of MDRO strain dissemination across species barriers in regional settings. Presence of ESBL-E and LA-MRSA among pets and probable dissemination in clinical settings support the necessity of a “One Health” approach to address the potential threats due to MDRO-carrying companion animals.

## Introduction

Today, companion animals such as cats and dogs are often considered family members and close proximity or direct animal contact are given on a daily basis in many households. This causes the potential risk of transmission of a multitude of pathogenic microorganisms, including multidrug-resistant bacteria, between pets and their human owners [1–3]. Coagulase-positive staphylococci, such as methicillin-resistant (MR) *Staphylococcus aureus* (MRSA), *Staphylococcus pseudintermedius* (MRSP) and *Staphylococcus intermedius* (MRSI), as well as MR coagulase-negative staphylococci (MR-CoNS) and extended-spectrum beta-lactamase (ESBL) producing *Enterobacteriaceae* (ESBL-E) have been shown to colonize companion animals and cause infections in both pets and humans [4–9].

The ability of bacteria to inhabit different hosts poses the additional threat of resistance gene transfer, resulting in decreased susceptibility against antimicrobials in increasing numbers of bacteria [4,10]. Resistance genes of particular interest comprise the *mec* genes (*mecA*, *mecB*, *mecC*, and *mecD*) causing methicillin resistance in members of the genera *Staphylococcus* and *Macrococcus*, and the *bla* genes (*bla*_CMY-2_, *bla*_SHV_, *bla*_TEM_, *bla*_CTX-M_) encoding beta-lactamases (especially ESBL) in *Enterobacterales* comprising *Enterobacteriaceae* and related families [11–16].

The aim of this study was to assess carriage and antimicrobial resistance of opportunistic animal and human pathogens in companion animals in North-West Germany by means of culture-based phenotypic and molecular methods.

## Materials and methods

### Sample collection

Bacterial isolates were obtained from dogs (n = 192), cats (n = 74), and rabbits (n = 17) either healthy or undergoing veterinary examination between May 2015 and March 2016. Animals treated in a private veterinary practice and a veterinary hospital or living in an animal shelter and private households were included. Swab samples (Transwab Amies MW 172P, Medical Wire & Equipment, Corsham Wiltshire, England) were collected from each animal’s nasal vestibules (both sides using one swab), oral mucosa and perianal area. Samples from animals in the hospital were taken prior to treatment. In the veterinary practice, animals were treated as outpatients. In order to participate in the study, pet owners had to answer a full consent form and a questionnaire. Samples were stored for a maximum of three days at room temperature before further processing.

### Cultivation

For isolation of *S*. *aureus*, nasal and buccal swabs were streaked onto selective chromogenic medium (chromID *S*. *aureus*, bioMérieux, Marcy l´Étoile, France), then suspended in 5 ml of tryptic soy broth (TSB, Becton Dickinson, Franklin Lakes, NJ, USA) supplemented with 6.5% NaCl. Solid and liquid cultures were incubated at 37°C for 24 h. Subsequently, chromID *S*. *aureus* agar was inoculated with 10 μl of the enriched liquid culture. Furthermore, 1 ml of the culture was used on chromogenic medium selective for MRSA (chromID MRSA, bioMérieux) and 1 ml was suspended in 9 ml phenolred mannitol broth supplemented with ceftizoxim/aztreonam (PHMB+C/AZ, Mediaproducts BV, Groningen, Netherlands). Cultures were grown at 37°C for either 24 h (chromID *S*. *aureus*, bioMérieux and PHMB+C/AZ, Mediaproducts BV) or 48 h (chromID MRSA, bioMérieux). From the PHMB+C/AZ (Mediaproducts BV) culture, 1 ml was used for further incubation on chromID MRSA (bioMérieux) at 37°C for 48 h.

For a subset of samples from 20 animals (13 dogs, 3 cats and 4 rabbits), which were the first animals included in the study, swabs were additionally streaked onto 5% sheep blood agar supplemented with aztreonam and colistin (CAP) (Oxoid, Wesel, Germany) and incubated at 37°C for 24 h for the detection of staphylococcal species apart from *S*. *aureus*.

In order to detect ESBL-E, swab samples from the perianal area were suspended in TSB and incubated at 37°C for 24 h. Chromogenic selective medium (chromID ESBL, bioMérieux) was inoculated with 10 μl of the liquid culture and further incubated at 37°C for 24 h.

### Identification of isolates

Colonies grown on chromID MRSA (bioMérieux), chromID *S*. *aureus* (bioMérieux), and CAP agar (Oxoid) were isolated based on conventional phenotypic characteristics, such as colony morphology, pigmentation, Gram staining and production of clumping factor (Pastorex Staph Plus; bioMérieux). Colonies grown on chromID ESBL medium (bioMérieux) were selected according to their colony morphology and pigmentation. Single colonies were inoculated onto Columbia blood agar (Becton Dickinson) and further cultivated at 37°C for 24 h.

Identification of pure cultures down to the species level was accomplished via matrix-assisted laser desorption/ionization-time-of-flight mass spectrometry (MALDI-TOF MS, Bruker Daltonics, Billerica, MA) as described elsewhere [17,18]. In brief, cells from freshly grown single colonies were smeared on a ground steel target plate (Bruker Daltonics) and covered with 1 μl of alpha-cyano-4-hydroxycinnamic acid (HCCA) dissolved in 50.0% acetonitrile and 2.5% trifluoroacetic acid. Analysis of co-crystallized samples was performed with flexControl 3.3 (Bruker Daltonics). Spectra were evaluated with the MALDI Biotyper 4.0 (Bruker Daltonics) considering an m/z range of 4,000–10,000 Da. The score threshold for explicit determination on species level was set to ≥ 2.0.

Bacterial isolates yielding scores ≤ 2.0 in MALDI-TOF MS analysis were subjected to biochemical identification in the VITEK 2 automated system (bioMérieux) using VITEK 2 GP ID cards for nasal and buccal samples or VITEK 2 GN ID cards (bioMérieux) for perianal samples, respectively.

If identification results via MALDI-TOF MS and VITEK 2 were ambiguous, the 16S rRNA gene was sequenced. For this, total genomic DNA was extracted using the QIAamp DNA MiniKit following the manufacturer’s instructions (Qiagen, Venlo, Netherlands). Primers 27f and 907r(m) were used to target the V1-V4 hypervariable regions of the 16S rRNA gene [19,20]. Purification of PCR products was achieved using the MinElute Kit (Qiagen) according to manufacturer’s instructions. Sequencing was performed on an ABI 3730XL sequencing machine using the cycle sequencing technology (Eurofins Genomics, Ebersberg, Germany). For sequence analysis, comparison against sequences obtained from validly described type strains and isolates was carried out using the Seqmatch function of the RDP-II database [21]. Sequences yielding a similarity score of ≥ 98% were assigned at species level.

### Characterization of isolates

Antimicrobial susceptibility testing (AST) of isolates identified as staphylococci or *Enterobacteriaceae* was carried out with the VITEK 2 system (bioMérieux) according to the manufacturer’s instructions using the test cards AST-P632 for staphylococci and AST-N214 for *Enterobacteriaceae*. AST results were interpreted according to The European Committee on Antimicrobial Susceptibility Testing (EUCAST) clinical breakpoints [22].

*S*. *pseudintermedius* and *S*. *intermedius* isolates were further characterized regarding their phenotypic resistance by disk diffusion using oxacillin (1 μg) as recommended by EUCAST [22]. The presence of the methicillin resistance determinants *mecA*, *mecB* and *mecC* in staphylococci was tested by PCR as previously described [11,23]. *S*. *aureus* isolates were genotyped by means of their *spa* gene and multilocus sequence typing (MLST) was performed on MRSA isolates as described elsewhere [24,25].

In *Enterobacteriaceae*, ESBL-production was confirmed using the MASTDISCS ID Extended-Spektrum-β-Laktamasen (ESβL)-Set (CPD10) D67C (MAST Diagnostica, Reinfeld, Germany) according to the manufacturer’s instruction. Resistance was further classified by detection of *bla*_SHV_, *bla*_TEM_ and *bla*_CTX-M_ genes by multiplex PCR as introduced by Monstein et al. [26]. The gene *bla*_CMY-2_ was tested as described by Souna et al. [27]. Additionally, the eazyplex SuperBug assay (AmplexDiagnostics GmbH, Gars am Inn, Germany) to detect carbapenemase-producing *Enterobacteriaceae* (CRE) was applied following the manufacturer’s instructions.

Association of ESBL-E/cefotaxime-resistant *Enterobacteriaceae* and MRSA carriage with potential risk factors was assessed separately for cats, dogs and rabbits applying Fisher’s exact test in GraphPad Prism v.5.00 (GraphPad Software, La Jolla, CA, USA); p < 0.05 was considered significant.

## Results

### Sample group

A total of 283 animals was sampled including 192 dogs, 74 cats and 17 rabbits. The mean age of the dogs was 5.6 years (range: 0.1–16 years) with 85 animals being male and 107 being female. Among the male dogs, 37.6% (n = 32) were castrated, 39.2% (n = 42) of female dogs were neutered. The group of cats had a mean age of 4.2 years (range: 0.1–17 years), the group of rabbits a mean age of 2.4 years (range 0.3–10 years). Among cats, 63.8% (23/36) of male cats were castrated and 55.3% (21/38) female cats were neutered. The gender distribution in the group of rabbits was nine male and eight female animals. Among these, 33.3% (3/9) of male and 25% (2/8) of female rabbits were neutered.

Across the complete group of animals, 31.4% (89/283) were healthy whereas 68.6% (194/283) were sampled before undergoing veterinary examinations. Altogether, antibiotics had been administered to 27.9% of animals (79/283) within six months prior to sampling. More details about the sample groups are provided in Table 1.

**Table 1:** Metadata of animals sampled.

Characteristics		Dogs	Cats	Rabbits	Total
Age (y)	Mean	5.6	4.2	2.4	5.1
	Range	0.1–16	0.1–17	0.3–10	0.1–17
Gender^a^	Male	85 (32)	36 (23)	9 (3)	130 (58)
	Female	107 (42)	38 (21)	8 (2)	153 (65)
Origin of sample	Animal shelter	4	17	0	21
	Private household	45	19	0	64
	Private veterinary practice	91	31	17	139
	Veterinary hospital	52	7	0	59
Veterinary examination	Yes	128	50	16	194
	No	64	22	1	89
Reason for veterinary examination^b^	Standard examination	47	18	14	79
	Internal diseases	20	19	0	39
	Surgical intervention	41	12	2	55
	Orthopedics	20	1	0	21
Antibiotic treatment^c^	Topical	9	5	0	14
	Systemic	41	19	0	60
	Both	2	2	1	5
	No antibiotics	140	48	16	204
Contact with other animals	Livestock	35	14	4	53
	Horses	79	19	0	98
	Dogs	78	23	2	103
	Cats	40	53	0	93
	Rodents	13	1	12	26
	Birds	1	1	0	2
Owner stayed abroad^c^		72	17	4	93
Total		192	74	17	283

^a^Numbers in brackets give total numbers of neutered animals

^b^Standard examination: general examination and consultation, vaccination, parasite prophylaxis, or dental cleaning. Internal diseases: diseases of digestive tract, urogenital tract, circulatory system, nervous system, skin, eyes, ears, or metabolism. Surgical intervention: Sterilization, wound management, orthopedic surgery, or tumor resection. Orthopedics: diagnosis of lameness, radiography, or bandage management.

^c^ within six months prior to sampling.

### Colonization with beta-lactam-resistant *Enterobacteriaceae*

Among all animals (n = 283), seven (2.5%) were found to carry ESBL-*Escherichia coli*; one sample was positive for cefotaxime-resistant *Citrobacter freundii* (Table 2). Whereas all *E*. *coli* isolates originated from the perianal area of dogs (7/192, 3.6%), the *C*. *freundii* isolate was detected in the perianal sample of a cat (1/74, 1.4%). All ESBL-E/cefotaxime-resistant *Enterobacteriaceae* isolates were susceptible to ertapenem, imipenem, meropenem and tigecycline. The assay for the detection of CRE remained negative. Four *E*. *coli* isolates harbored single *bla* genes (*bla*_TEM_: n = 1; *bla*_CTX-M_: n = 3), while four isolates carried combinations of *bla* genes (*C*. *freundii*: *bla*_TEM_ and *bla*_CMY-2_: n = 1; *E*. *coli*: *bla*_TEM_ and *bla*_SHV_: n = 1, *bla*_TEM_ and *bla*_CTX-M_: n = 2) (Table 2).

**Table 2:** Resistance profiles of cefotaxime-resistant *Enterobacteriaceae* found in 283 companion animals.

Species	Animal host^a^	Sampling site	Resistance genes	Phenotypic antimicrobial susceptibility test profile	
				ESBL-Screening^b^	Other resistances^c^
*Citrobacter freundii*	Cat (#2)	Perianal	*bla*_TEM_, *bla*_CMY-2_	NEG	AMP, SAM, TZP, CXM, CPD, CTX, CAZ
*Escherichia coli*	Dog (#121)	Perianal	*bla*_CTX-M_	POS	AMP, SAM, CXM, CPD, CTX, SXT
	Dog (#130)	Perianal	*bla*_TEM_	POS	AMP, SAM, CXM, CPD, CTX
	Dog (#146)	Perianal	*bla*_TEM_, *bla*_SHV_	POS	AMP, SAM, CPD, CAZ, MXF, SXT
	Dog (#147)	Perianal	*bla*_CTX-M_	POS	AMP, SAM, CXM, CPD, CTX, CAZ, SXT
	Dog (#160)	Perianal	*bla*_TEM_, *bla*_CTX-M_	POS	AMP, SAM, CXM, CPD, CTX, CIP, MXF
	Dog (#163)	Perianal	*bla*_CTX-M_	POS	AMP, SAM, CXM, CPD, CTX, CIP, MXF, SXT
	Dog (#182)	Perianal	*bla*_TEM_, *bla*_CTX-M_	POS	AMP, SAM, CXM, CPD, CTX

^a^ Individual running numbers of animals are given in brackets.

^b^ as determined by MASTDISC ID ESβL-Set (CPD10) D67C (MAST Diagnostica)

^c^ MICs were detected with VITEK 2 (bioMérieux) and evaluated using breakpoints provided by EUCAST [22].

POS, positive; AMP, ampicillin; CAZ, ceftazidime; CIP, ciprofloxacin; CPD, cefpodoxime; CTX, cefotaxime; CXM, cefuroxime; MXF, moxifloxacin; SAM, ampicillin-sulbactam; SXT, trimethoprim-sulfamethoxazol; TZP, piperacillin-tazobactam.

The dogs colonized with ESBL-producing *E*. *coli* were 2–12 years old (mean: 7.7 years). Five dogs were male, the other two were female, all of which were neutered except for one male dog. All seven animals were undergoing veterinary examination either in a private veterinary practice (2/7) or a veterinary clinic (5/7). Reasons for the examination were internal diseases (4/7), orthopedics (2/7), or surgical interventions (1/7). Antibiotics had been administered to 4/7 dogs within six months before sampling. One dog had regular contact with livestock and other companion animals, three had contact with horses and companion animals, two had contact with other companion animals and one was not kept together with any other animals. Positive ESBL-*E*. *coli* samples from dogs admitted to the veterinary clinic (5/7) were detected within a short time interval between 01/2016 and 03/2016.

The cat carrying cefotaxime-resistant *C*. *freundii* was 0.5 years old, male and not neutered. It was brought into the private veterinary practice for surgical intervention and had not received any antibiotics prior to sampling. It was reported to live together with another cat and a dog.

Amongst the samples obtained from rabbits, no ESBL-E were detected.

### Colonization with *S*. *aureus*

Twenty of 192 dogs (10.4%) carried *S*. *aureus*. Colonizing strains (n = 26) belonged to *spa* types t008, t034 (each n = 4), t091 (n = 3), t605, t786, t3750 (each n = 2), t002, t011, t019, t108, t275, t620, t630, t16020, and t16021 (each n = 1). In 11/20 animals, *S*. *aureus* was exclusively detected in the nose (55%). five animals (25%) were tested positive only in their mouth. The remaining four animals carried *S*. *aureus* in both habitats. In these four animals, isolates from nasal and buccal samples always belonged to the same *spa* type (t034, t605, t786 and t3750). In the nose of one dog, isolates associated with three different *S*. *aureus spa* types (t008, t091 and t620) were found. Methicillin resistance conferred by the gene *mecA* was found in six *S*. *aureus* isolates colonizing 5/192 dogs (2.6%). These isolates belonged to *spa* types t034 (n = 4), t011 and t108 (each n = 1), all associated with sequence type (ST) 398. All canine MRSA isolates (6/6) showed resistance against tetracycline (Table 3). MRSA positive animals were of variable age (0.2–7 years, mean 3.6 years) and gender (3 female, 2 male) and were either admitted to the private practice (n = 4) or the clinic (n = 1). One of the dogs was a healthy animal, accompanying another animal treated by the veterinarian. The other dogs were admitted to the veterinarian for standard examination (2/5) or surgical intervention (2/5). Among the the MRSA positive dogs, 1/5 were reported to have contact with livestock, 1/5 had contact with other companion animals, such as other dogs, cats, or rabbits, and 3/5 had contact with both horses and companion animals. Systemic antibiotics had been administered to 2/5 dogs.

**Table 3:** Resistance profiles of (i) methicillin-resistant *Staphylococcus aureus* (MRSA) from 283 companion animals and (ii) methicillin-resistant coagulase-negative staphylococci from 20 companion animals.

Species	Animal host^a^	Sampling site	*spa* type	Resistance genes	Phenotypic antimicrobial susceptibility test profile	
					FOX screening^b^	Other resistances^c^
*Staphylococcus aureus*	Dog (#12)	Nasal	t108	*mecA*	POS	PEN, OXA, TET
	Dog (#16)	Nasal	t034	*mecA*	POS	PEN, OXA, CLI, ERY, TET
	Dog (#89)	Buccal	t011	*mecA*	POS	PEN, OXA, LVX, TET
	Dog (#103)	Nasal	t034	*mecA*	POS	PEN, CLI, TET
	Dog (#171)	Buccal	t034	*mecA*	POS	PEN, OXA, LVX, TET, SXT
	Dog (#171)	Nasal	t034	*mecA*	POS	PEN, OXA, LVX, TET, SXT
	Cat (#44)	Buccal	t843	*mecC*	POS	PEN, OXA
	Cat (#44)	Nasal	t843	*mecC*	POS	PEN
	Cat (#67)	Buccal	t011	*mecA*	POS	PEN, OXA, LVX, TET
*Staphylococcus cohnii* subsp. *cohnii*	Dog (#2)	Buccal		*mecA*	POS	OXA, FOF
*Staphylococcus cohnii* subsp. *urealyticus*	Rabbit (#3)	Nasal		*-*	POS	OXA, FOF, FA
	Rabbit (#3)	Buccal		*-*	POS	OXA, FOF, FA
*Staphylococcus pettenkoferi*	Rabbit (#1)	Buccal		*-*	POS	OXA, FOF
*Staphylococcus saprophyticus* subsp. *saprophyticus*	Rabbit (#3)	Nasal		*mecA*	POS	OXA, FOF, FA

^a^ Individual running numbers of animals are given in brackets.

^b^ as determined by VITEK 2 automated system (bioMérieux).

^c^ MICs were detected with VITEK 2 (bioMérieux) and evaluated using breakpoints provided by EUCAST [22].

POS, positive; CLI, clindamycin; ERY, erythromycin; FOF, fosfomycin; FA, fusidic acid; LVX, levofloxacin; OXA, oxacillin; PEN, penicillin; TET, tetracycline; SXT, trimethoprim-sulfamethoxazol.

In the group of 74 cats, six (8.1%) were characterized as *S*. *aureus* carriers. Isolates (n = 9) were assigned to *spa* types t843 (n = 3), t011 and t11232 (each n = 2), and t015 and t1736 (each n = 1). In two of these cats, *S*. *aureus* was found only in the nose, in another two only in the mouth and in the remaining two in both habitats. Isolates colonizing different habitats in the same animal were shown to be of the same *spa* types (t843 and t11232). Three *S*. *aureus* isolates colonizing 2/74 animals (2.7%) were MR. Of these, one isolate (*spa* type t011, ST398) carried *mecA*, whereas the other two isolates, both *spa* type t843 and ST130 obtained from mouth and nose of the same animal, carried *mecC*. The *mecA-*carrying isolate showed further resistances against clindamycin, levofloxacin and tetracycline (Table 3). The two cats carrying MRSA were eight and ten years old, both female and neutered and both brought to the veterinarian practice because of internal diseases. Neither of them was reported having contact to livestock or horses, but both were kept together with other pets, such as dogs and other cats. The cat carrying the *mecA-positive* MRSA had received systemic and local antibiosis within six months prior to sampling. The animal carrying the *mecC-*positive t843 isolates in mouth and nose also carried another nasal t843 *S*. *aureus* strain which was methicillin-susceptible according to phenotypic and molecular tests.

Amongst the rabbits, no *S*. *aureus*/MRSA isolates were found.

### Association of risk factors with MDRO carriage

In the group of dogs, a linkage between neutered animals and ESBL-*E*. *coli* carriage was shown (6/68 vs. 1/117; p = 0.0138). None of the other assessed risk factors was associated with ESBL-E/cefotaxime-resistant *Enterobacteriaceae* or MRSA carriage in the three groups of animals (p > 0.05 for all variables). However, when analyzing ESBL-E/cefotaxime-resistant *Enterobacteriaceae* and MRSA (*i*.*e*. MDRO) together and carriage among all tested animals (not separately for different species), we found that animals sampled in practices and hospitals carried MDRO more frequently than animals sampled in shelters and households (17/181 vs. 0/85; p = 0.0023). Moreover, veterinary treatment (16/178 vs. 1/88; p = 0.0161), castration (12/111 vs. 5/155; p = 0.0238) and contact to other pets (15/163 vs. 2/103; p = 0.0355), were associated with MDRO carriage.

### Colonization with staphylococci other than *S*. *aureus*

**Fig 1:**
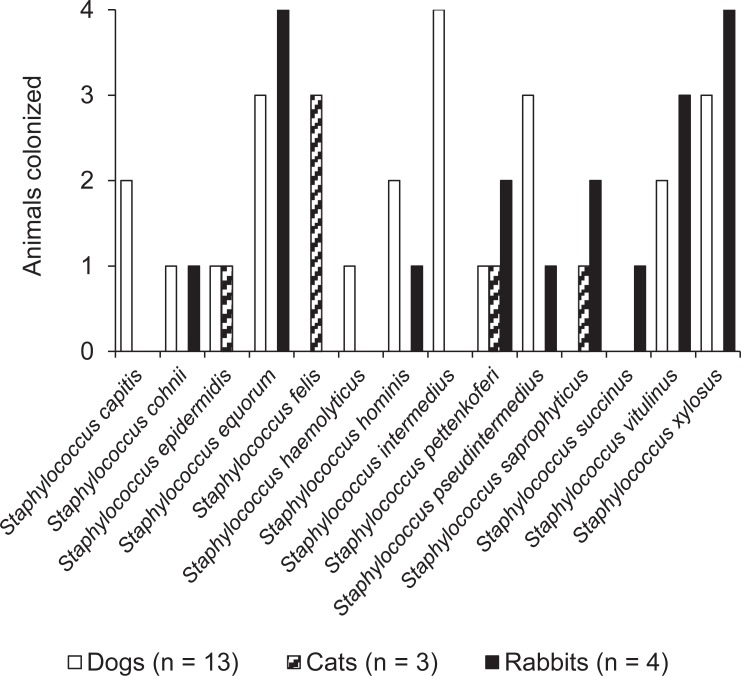
Absolute numbers of companion animals (dogs, cats, rabbits) colonized with different staphylococcal species other than *S*. *aureus* in the subgroup of 20 animals.

A subgroup of animals (n = 20), consisting of 13 dogs, three cats and four rabbits was further analyzed for staphylococcal species other than *S*. *aureus*. Altogether, 14 different staphylococcal species were detected across this subgroup (Fig 1).

In the group of dogs, eleven different species were detected with the coagulase-positive species *S*. *intermedius* (in 4/13 dogs; 30.8%) and *S*. *pseudintermedius* (in 3/13 dogs; 23.1%) and the CoNS *Staphylococcus equorum* and *Staphylococcus xylosus* (both in 3/13 dogs; 23.1%) being most prevalent. Amongst the cats, four different staphylococcal species were detected. In this group, *Staphylococcus felis* was found in 3/3 of cats whereas the remaining three species were found in 1/3 cats each. Throughout the group of rabbits, nine different staphylococcal species were detected, with *S*. *equorum* and *S*. *xylosus* both being detected in 4/4 of the rabbits. Other prevalent species were *Staphylococcus vitulinus* (in 3/4 rabbits), *Staphylococcus pettenkoferi* and *Staphylococcus saprophyticus* (each in 2/4 rabbits) (Fig 1).

Screenings for methicillin resistance among staphylococcal isolates revealed one *Staphylococcus cohnii* subsp. *cohnii*, two *Staphylococcus cohnii* subsp. *urealyticus*, one *S*. *pettenkoferi*, and one *Staphylococcus saprophyticus* subsp. *saprophyticus* isolate showing phenotypic resistance according to VITEK 2 testing. Presence of the gene *mecA* was verified for one MR-*S*. *cohnii* subsp. *cohnii* isolate in a dog and one MR-*S*. *saprophyticus* subsp. *saprophyticus* in a rabbit (Table 3). The animals carrying these two strains had not been in direct contact with livestock or horses but were both in contact with other companion animals. Moreover, the dog carrying MR-*S*. *cohnii* subsp. *cohnii* had received topical antibiotics before sampling and was at the veterinary practice for a surgical intervention. The rabbit carrying MR-*S*. *saprophyticus* subsp. *saprophyticus* had been brought to the veterinarian practice for a standard examination.

## Discussion

The risk of zoonotic transmission of bacteria between animals and humans is a global problem challenging human and veterinary medicine and beyond, such as food industry and wildlife, which necessitates inter- and transdisciplinary linking within a One Health concept [1,5,28,29]. However, most studies focus on the analysis of zoonotic agents in livestock whereas comprehensive data on the colonization of companion animals are limited. In contrast to livestock, companion animals are often considered as family members living in very close contact with their owners. Thus, transmission between humans and animals in both directions is very likely [3]. This study gives an overview of the prevalence of ESBL-E and MRSA in companion animals. Moreover, it provides insights into the occurrence of other staphylococcal species constituting potential reservoirs for methicillin resistance determinants.

In this point-prevalence assessment, we found that the proportion of animals colonized with ESBL-*E*. *coli* (2.5% of all animals or 3.6% of the dogs) was comparable to other studies investigating the perianal/rectal carriage of companion animals [30–32]. On the other hand, consecutive screenings of feline and canine fecal samples revealed considerably higher abundances of ESBL-E [33,34], showing the different outputs generated by the two approaches. Naturally, perianal swab samples only yield small amounts of fecal material, resulting in the recovery of only a fraction of bacterial isolates when compared to the corresponding fecal sample. Nevertheless, we deliberately chose this approach in order to efficiently collect and maximize the number of samples. Moreover, a swab sample of the perianal area provides valuable information about the risk of ESBL-E transmission from pet to owner, as the animals tested positive in this habitat present a permanent risk factor for the people in close contact.

The prevalence of MRSA among cats and dogs was generally lower than described for livestock [35,36]. However, it corresponded with carriage rates reported for humans in the general German population [37–39]. Previous studies found similar data in companion animals (0.1–5.7%) [40–43]. Interestingly, all MRSA isolates found were linked to *spa* types belonging to the livestock-associated ST398 (t011, t034, t108) and ST130 (t843), respectively. This is particularly interesting, as the majority of these animals (6/7) did not live in direct contact with livestock. However, the samples of this study were obtained from animals living in rural districts in North-West Germany, an area well known for its high density of husbandry, in particular pig farming. In this area, a significant increase of LA-MRSA, particularly CC398, into human healthcare facilities has been observed over the last decades [44,45]. Accordingly, the high colonization rate of this clonal lineage in companion animals could reflect the occurrence of LA-MRSA in the general and hospitalized human population of this area [37,45,46]. Moreover, Bierowiec et al. (2016) demonstrated that the prevalence of *S*. *aureus* is significantly higher in domestic cats than in feral cats [47]. This underpins previous assumptions of a bacterial ‘spill over’ from owners to their pets, thus creating a reservoir for (re-)colonization and infection [5,48–50]. The findings of this study are also in line with data about epidemic extended-host-spectrum MRSA lineages, which instead of showing host specificity can rather be assigned to a specific geographic origin [10,51]. Moreover, it has been shown, that especially MRSA show a high potential to spread via dust and air into the closer farm environment [52, 53]. Apart from this, the fact that all MDROs were either obtained from one veterinary practice or one veterinary clinic, strongly suggest a nosocomial spread of these strains. A significant association of these factors could be demonstrated (Fisher’s exact test; p < 0.05). This finding is further substantiated by the close relation of LA-MRSA *spa* types found and the very short time interval where ESBL-*E*. *coli* were detected particularly in animals treated ambulatory in the veterinary clinic. A general rise in veterinary nosocomial outbreaks for different MDROs has been well-documented in recent years [5,54,55]. Again, the ‘spill over’ from veterinary personnel, other animal patients and their human owners, and the distinct geographic origin would be key factors for such an outbreak.

Other coagulase-positive staphylococcal species, also often described as the *S*. *aureus* counterparts in veterinary medicine, are *S*. *intermedius* and *S*. *pseudintermedius*. Both species are well-known for causing different types of pyogenic and skin infections, such as pyoderma, dermatitis and otitis in small companion animals, particularly in dogs [56–61]. Moreover, the prevalence of MR strains has been increasing rapidly, leading to significant health problems in animals, particularly in veterinary hospital settings [62–65]. In the subgroup of 20 animals analyzed for the colonization of different staphylococcal species, *S*. *pseudintermedius* was found in dogs (3/13) and rabbits (1/4), but not in cats. *S*. *intermedius* was found only in dogs (4/13). However, none of these strains showed resistance against methicillin.

Apart from the coagulase-positive species *S*. *intermedius* and *S*. *pseudintermedius*, another twelve different coagulase-negative staphylococcal species were shown to be present in the subset of 20 animals. Amongst these, one canine *S*. *cohnii* subsp. *cohnii* isolate and one *S*. *saprophyticus* subsp. *saprophyticus* isolate in a rabbit were shown to be phenotypically MR and to harbor the *mecA* gene. In humans, *S*. *saprophyticus* subsp. *saprophyticus* is well known as a frequent cause for urinary tract infections in young women [66–71]. In animals, *S*. *saprophyticus* has been described as a colonizer of the gastrointestinal tract of cattle and pigs, a causative agent for subclinical mastitis in cows, and as a contaminant of animal food products [72–74]. Less data are available on *S*. *cohnii* subsp. *cohnii*, which is described as a colonizer of dogs, goats and poultry, but has also been detected in the hospital environment and in clinical samples of humans suffering from a variety of infectious diseases [66,75–77]. In the past decades, the prevalence of MR-CoNS is rapidly increasing, reaching levels of up to over 80% within clinical samples of human patients [66,78–80]. In the animal cohort investigated here, at least 10.0% of animals (2/20) were colonized with MR-CoNS. Another two animals were colonized with other phenotypically resistant staphylococcal strains. These strains might either serve as a reservoir of methicillin resistance genes for other zoonotic pathogens or could be transferred directly to humans, such as pet owners or veterinary personnel.

## Conclusion

Data of this study prove that resistances against beta-lactams and other antibiotic classes are ubiquitously present in medically significant pathogens colonizing companion animals. This situation constitutes a high risk of transmission of MDROs or resistance-encoding mobile elements to humans being in close contact to those animals and their microbiota. The exclusive occurrence of only those MRSA belonging to livestock-associated clonal lineages in companion animals without direct contact to livestock emphasizes the adverse effects of MDRO dissemination across species barriers in regions with high density of livestock husbandry. The findings underline the need for the consequent implementation of the One Health concept considering all interconnections between human and animal populations where pathogens and their resistance mechanisms can be transmitted.
